# Síntomas subagudos y crónicos de la fiebre de chikungunya en un grupo de personas adultas en Ibagué, Colombia

**DOI:** 10.7705/biomedica.4350

**Published:** 2019-09-01

**Authors:** Juan Sebastián Sánchez, Ana María Cañón, Jadith Cristina Lombo

**Affiliations:** 1 Programa de Enfermería, Facultad de Ciencias de la Salud, Universidad del Tolima, Ibagué, Colombia Universidad del Tolima Programa de Enfermería Facultad de Ciencias de la Salud Universidad del Tolima Ibagué Colombia

**Keywords:** Aedes, fiebre de chikungunya/epidemiologia, signos y síntomas, artralgia, Colombia, Aedes, chikungunya fever/epidemiology, signs and symptoms, arthralgia, Colombia

## Abstract

**Introducción:**

. La fiebre de chikungunya en Colombia ocasiona una alerta en salud pública que se extiende en el tiempo, dadas las características subagudas y crónicas de la enfermedad.

**Objetivo.:**

Describir los síntomas subagudos y crónicos en personas mayores de 18 años con fiebre de chikungunya en Ibagué, Colombia.

**Materiales y métodos.:**

Se hizo un estudio descriptivo con muestreo en bola de nieve de 368 adultos de todas las comunas de Ibagué que presentaban síntomas de la fiebre de chikungunya de más de 12 días de duración.

**Resultados.:**

Las articulaciones que presentaban dolor en la fase subaguda y crónica de la enfermedad fueron las de manos (84,2 %), rodillas (72,8 %) y tobillos (69,3 %); además de las articulaciones, la planta de los pies fue el sitio en que más frecuentemente se presentó dolor (48,8 %). El dolor articular en manos (p=0,017) y tobillos (p=0,001) y el dolor en la planta de los pies (p=0,002) fueron significativos en las mujeres. La fatiga o el cansancio generalizado se presentaron en el 58,9 % de las personas y se prolongó por más de un año en el 2,4 % de ellas.

**Conclusiones.:**

Los síntomas de la fase subaguda y crónica de la fiebre de chikungunya coincidieron con los descritos en la literatura médica, su duración se extendió hasta por un año o más en algunos de los casos y su intensidad disminuyó con el tiempo. Los síntomas referidos fueron más comunes en las mujeres que en los hombres.

## Abstract (en)

**Introduction:**

In Colombia, chikungunya fever creates a warning in public health that extends over time given the subacute and chronic characteristics of the disease.

**Objective:**

To describe subacute and chronic symptoms in adult people who suffered chikungunya fever in the city of Ibagué, Colombia.

**Materials and methods:**

We conducted a descriptive study with snowball sampling in 368 adults from all the communes of Ibagué, Colombia, who had symptoms of chikungunya fever spanning for more than 12 days.

**Results:**

The joints that showed the greatest pain in the subacute and chronic phase of the disease were the hands with 84.2%, knees with 72.8%, and ankles with 69.3%. The soles of the feet were the non-articular areas with greater frequency of pain (48.8%). Joint pain in the hands (p=0.017) and ankles (p=0.001) and pain in the soles of the feet (p=0.002) were significant in women. General fatigue occurred in 58.9% of the subjects and in 2.4% of the population, it lasted for more than a year.

**Conclusions:**

The symptoms of the subacute and chronic phase of chikungunya fever were the same reported in the literature, they lasted up to a year or more, and decreased in intensity over time. The referred symptoms were more common in women than in men.

La fiebre de chikungunya es una enfermedad viral que se transmite al ser humano por los zancudos *Aedes aegypti y Aedes albopictus*. El virus chikungunya es un virus ARN del género *Alfavirus*, familia Togaviridae, que históricamente ha generado brotes de grandes proporciones en África, Asia y Europa, por lo que desde el 2010 se consideró que podía producir una epidemia emergente ^1^.

Entre el 2014 y el 2016, el Instituto Nacional de Salud de Colombia reportó un total de 484.684 personas con fiebre de chikungunya, de las cuales 479.817 fueron confirmadas por clínica y 4.867 por laboratorio ^2^^,^^3^. Desde la semana epidemiológica 01 del 2015 a la 26 del 2016, se reportaron en Ibagué 12.317 personas con fiebre de chikungunya, de las cuales 12.223 fueron confirmadas por clínica y 94 por laboratorio ^4^^,^^5^.

La fiebre de chikungunya se caracteriza por la aparición súbita de fiebre, generalmente acompañada de dolores articulares. Otros signos y síntomas frecuentes son los dolores musculares y de cabeza, las náuseas, el cansancio y las erupciones cutáneas. Los dolores articulares suelen ser muy debilitantes, pero generalmente desaparecen en pocos días. A menudo las personas infectadas con el virus solo presentan sintomatología leve sin mayores complicaciones; entre el 3 y el 28 % de los casos son asintomáticos, pero contribuyen a la propagación de la enfermedad.

En los pacientes sintomáticos, la enfermedad atraviesa por tres fases: aguda, subaguda y crónica. La fase aguda dura entre 3 y 12 días y se caracteriza por presentar un pico febril de más de 39 °C, es de comienzo súbito y produce artralgias intensas; pueden presentarse cefaleas, mialgias, fatiga, náuseas, vómito y conjuntivitis. Los síntomas relacionados con el sistema osteomuscular son simétricos en manos y pies, y se acompañan de inflamación. Con la disminución de la fiebre, a los dos o tres días, se observa exantema máculo-papular en tronco y extremidades. La fase subaguda tiene una duración de 12 a 90 días y se caracteriza por la continuidad de los síntomas; en algunos pacientes hay exacerbación del dolor articular, tenosinovitis de muñecas y tobillos y fatiga. La fase crónica se caracteriza por síntomas que han persistido durante más de tres meses y pueden prolongarse hasta por un año o más. Esta fase es más frecuente en los grupos de riesgo, como quienes están en los extremos de la edad, y aquellos que sufren enfermedades de base ^6^^-^^7^.

En diversos estudios en los países africanos, en India y en Asia ^8^^-^^9^, el curso clínico de la fase aguda de la fiebre de chikungunya ha sido bien establecido y, después de terminada esta fase, en las fases subaguda y crónica, la evolución de la enfermedad se caracteriza por artralgia y artritis reumatoide persistentes, respectivamente. En dichos estudios, se plantea la necesidad de caracterizar más ampliamente los síntomas posteriores a la fase aguda, pues se ha encontrado que los pacientes mejoran en forma adecuada con los analgésicos habituales, posiblemente porque el neurotropismo del virus del chikungunya hace que el dolor sea, no solo nociceptivo, sino también neuropático.

Dado que los síndromes de dolor neuropático requieren un tratamiento específico, sería necesario caracterizarlos mejor, incluida la detección de las características neuropáticas mediante medios clínicos fáciles de usar, pues ello podría contribuir a mejorar significativamente los resultados del tratamiento. En este contexto, en el presente estudio se planteó describir los síntomas subagudos y crónicos en personas mayores de 18 años con fiebre de chikungunya en Ibagué.

## Materiales y métodos

Se hizo un estudio descriptivo observacional en una población de 4.646 personas con diagnóstico clínico de fiebre de chikungunya atendidas en instituciones de salud públicas y privadas de Ibagué durante la semana epidemiológica 17 del 2015. Se calculó una muestra de 355 a 391 sujetos en Epidat con el intervalo de confianza (IC) de 95 % establecido para encuestas transversales en poblaciones finitas.

Después de aplicar los criterios de inclusión, se logró recolectar la información de 368 pacientes seleccionados mediante un muestreo en bola de nieve por conveniencia; en ninguna de las bases de datos se había discriminado el diagnóstico según fase subaguda y crónica, ya que las personas suelen consultar a los servicios de salud durante la fase aguda de la enfermedad, y no en las fases subaguda y crónica.

Los criterios de inclusión del estudio fueron: haber presentado síntomas relacionados con la fiebre de chikungunya durante 12 días o más, estar en la fase subaguda o en la crónica; haber presentado durante la fase aguda tres de los cuatro signos y síntomas característicos de la fiebre de chikungunya descritos en la literatura médica (fiebre mayor o igual a 39 °C, dolor articular muy intenso, inflamación en manos o pies y exantema maculopapular en tronco o extremidades) y, por último, tener 18 años de edad o más.

Teniendo en cuenta que Ibagué es un lugar endémico para dengue y zika, cuyas sintomatologías son similares a la de la fiebre de chikungunya, se establecieron los siguientes criterios de exclusión: haber presentado dolor retroocular o abdominal (síntomas característicos del dengue) o conjuntivitis (síntoma característico del zika).

La información se recolectó mediante una entrevista semiestructurada y ajustada después de la prueba piloto. Se incluyeron los datos sociodemográficos, los síntomas manifestados en las fases de la enfermedad, y el dolor evaluado mediante la escala análoga de dolor ^10^ con base en los criterios de la *International Association for the Study of Pain* (IASP) y de múltiples estudios que han reportado el dolor neuropático en la fibre de chikungunya ^8^^,^^11^^-^^12^.

Consideraciones éticas

Se tuvieron en cuenta los principios de la Declaración de Helsinki y la Resolución 8430 de 1993 expedida por el Ministerio de Salud de Colombia, por la cual se establecen las normas científicas, técnicas y administrativas para la investigación en salud. Cada paciente dio su consentimiento por escrito para participar en el estudio y los investigadores garantizaron la confidencialidad de los datos con el anonimato de la identificación según las normas del *habeas data*.

## Resultados

El 29,9 % de la muestra correspondía a hombres y el 70,1 % a mujeres; la edad media fue de 39,7 años con un rango de 18 a 80 años. El dolor articular se presentó en el 100 % de los casos, la fiebre de 39 °C o más, en el 98,1 %, el exantema máculo-papular, en el 88,6 %, y la inflamación de manos y pies, en el 85,3 % de la población estudiada. El dolor más prevalente fue el articular en las manos (84 %), seguido por el dolor en rodillas (72,8 %) y tobillos (69,3 %) (cuadro 1). El 44 % de los pacientes también manifestaron experimentar dolor en la planta de los pies, y 18, 2 % en los músculos de los brazos y en la cabeza (cuadro 1).

**Table 1:** Distribución de la población según la duración del dolor en cada sitio anatómico

Dolor		Fases					Total	χ**2**
		Subaguda	Crónica					
		>12 días <3 meses	≥3 meses <6 meses	≥6 meses <9 meses	≥9 meses <12 meses	≥12 meses		
Articular en manos	n	165	89	29	17	10	310	0,1356
	%	44,8	24,2	7,9	4,6	2,7	84,2	
Articular en codos	n	61	29	10	6	6	112	0,0036
	%	16,6	7,9	2,7	1,6	1,6	30,4	
Articular en hombros	n	65	30	8	5	6	114	0,1031
	%	17,7	8,2	2,2	1,4	1,6	31	
Articular en cadera	n	65	17	5	5	2	94	0,0001
	%	17,7	4,6	1,4	1,4	0,5	25,5	
Articular en rodillas	n	131	75	31	17	14	268	0,645
	%	35,6	20,4	8,4	4,6	3,8	72,8	
Articular en tobillos	n	122	72	36	13	12	255	0,394
	%	33,2	19,6	9,8	3,5	3,3	69,3	
Otras articulaciones	n	15	11	1	6	0	33	0,5931
	%	4,1	3	0,3	1,6	0	9	
Cabeza	n	47	14	6	0	0	67	0,0005
	%	12,8	3,8	1,6	0	0	18,2	
Muscular en brazos	n	41	14	7	3	2	67	0,0523
	%	11,1	3,8	1,9	0,8	0,5	18,2	
Muscular en espalda	n	27	6	4	2	0	39	0,0126
	%	7,3	1,6	1,1	0,5	0	10,6	
Muscular en muslos	n	43	10	4	3	1	61	0,0008
	%	11,7	2,7	1,1	0,8	0,3	16,6	
Muscular en piernas	n	39	17	6	3	0	65	0,09
	%	10,6	4,6	1,6	0,8	0	17,7	
Muscular en otro	n	17	2	2	2	0	23	0,0198
	%	4,6	0,5	0,5	0,5	0	6,3	
Planta de los pies	n	90	28	24	13	8	163	0,1313
	%	24,5	7,6	6,5	3,5	2,2	44,3	
Fatiga o cansancio	n	132	46	17	9	9	216	0,0000
generalizado	%	35,9	12,5	4,6	2,4	2,4	58,6	

Los dolores se presentaron en las dos fases, aunque más en la fase subaguda que en la crónica, observándose una tendencia lineal descendente (cuadro 2). Los dolores articulares del tronco superior (manos, codos, hombros) fueron predominantes en la fase subaguda y, los del tronco inferior, las rodillas, los tobillos y otras articulaciones, en la fase crónica (p>0,05) (cuadro 1). El dolor de cabeza y los dolores musculares predominaron en la fase aguda, con una diferencia significativa (p<0,05) frente a la crónica, aunque sin diferencias según su ubicación en los músculos del tronco inferior o superior.

**Cuadro 2:** Caracterización del dolor en articulaciones y otros sitios anatómicos

Dolor		Caracterización del dolor																
		Localización			Curso			Neuropático					Intensidad del dolor de 1 a 10					Total
		Localizado	Difuso	Total	Continuo	Discontinuo	Total	Hormigueo	Punzante	Quemante	Otro	Total	1-2	3-4	5-6	7-8	9-10	
Articular en manos	n	256	54	310	148	162	310	68	191	26	25	310	16	46	90	112	46	310
	%	82,6	17,4	100	47,7	52,3	100	21,9	61,6	8,4	8,1	100	5,2	14,8	29	36,1	14,8	100
Articular en codos	n	95	17	112	58	54	112	18	76	9	9	112	8	13	36	43	12	112
	%	84,8	15,2	100	51,8	48,2	100	16,1	67,9	8	8	100	7,1	11,6	32,1	38,4	10,7	100
Articular en hombros	n	85	29	114	67	47	114	9	84	8	13	114	6	18	40	39	11	114
	%	74,6	25,4	100	58,8	41,2	100	7,9	73,7	7	11,4	100	5,3	15,8	35,1	34,2	9,6	100
Articular en cadera	n	54	40	94	52	42	94	11	64	8	11	94	3	11	31	34	15	94
	%	57,4	42,6	100	55,3	44,7	100	11,7	68,1	8,5	11,7	100	3,2	11,7	33	36,2	16	100
Articular en rodillas	n	228	40	268	123	145	268	22	195	24	27	268	9	29	74	106	50	268
	%	85,1	14,9	100	45,9	54,1	100	8,2	72,8	9	10,1	100	3,4	10,8	27,6	39,6	18,7	100
Articular en tobillos	n	217	38	255	119	136	255	27	186	18	24	255	7	29	77	97	45	255
	%	85,1	14,9	100	46,7	53,3	100	10,6	72,9	7,1	9,4	100	2,7	11,4	30,2	38	17,6	100
Cabeza	n	39	28	67	20	47	67	5	52	6	4	67	3	10	20	22	12	68
	%	58,2	41,8	100	29,9	70,1	100	7,5	77,6	9	6	100	4,5	14,9	29,9	32,8	17,9	100
Muscular en brazos	n	37	30	67	40	27	67	9	35	15	8	67	2	14	25	19	7	67
	%	55,2	44,8	100	59,7	40,3	100	13,4	52,2	22,4	11,9	100	3	20,9	37,3	28,4	10,4	100
Muscular en espalda	n	22	17	39	25	14	39	2	21	13	3	39	0	8	13	13	5	39
	%	56,4	43,6	100	64,1	35,9	100	5,1	53,8	33,3	7,7	100	0	20,5	33,3	33,3	12,8	100
Muscular en muslos	n	33	28	61	37	24	61	4	31	14	12	61	2	11	23	20	5	61
	%	54,1	45,9	100	60,7	39,3	100	6,6	50,8	23	19,7	100	3,3	18	37,7	32,8	8,2	100
Muscular en piernas	n	38	27	65	41	24	65	7	32	13	13	65	1	12	27	19	6	65
	%	58,5	41,5	100	63,1	36,9	100	10,8	49,2	20	20	100	1,5	18,5	41,5	29,2	9,2	100
Muscular en otro sitio	n	13	10	23	17	6	23	1	9	11	2	23	1	6	8	7	1	23
	%	56,5	43,5	100	73,9	26,1	100	4,3	39,1	47,8	8,7	100	4,3	26,1	34,8	30,4	4,3	100
Planta de los pies	n	128	35	163	90	73	163	18	103	28	14	163	6	26	42	62	27	163
	%	78,5	21,5	100	55,2	44,8	100	11	63,2	17,2	8,6	100	3,7	16	25,8	38	16,6	100

El dolor neuropático punzante predominó más en las articulaciones, especialmente las de los hombros (73,7 %). El dolor descrito como una sensación de hormigueo fue el segundo en frecuencia en la mayoría de las articulaciones, principalmente las de las manos (21,9 %). La percepción del dolor articular medido con la escala análoga numérica fluctuó entre 7 y 8, con excepción del dolor en la cadera, presente en 35,5 % de las personas, con una puntuación entre 5 y 6. Las articulaciones con mayor calificación del dolor (9 a 10) fueron las rodillas y los tobillos (cuadro 2).

En los sitios anatómicos no articulares, el dolor localizado fue el más común, con el porcentaje más alto (78,5 %) para el dolor plantar, en tanto que el más bajo (55,2 %) se presentó en los músculos de los brazos. La población manifestó haber experimentado dolor muscular continuo en el 73,9 % de los sitios anatómicos estudiados.

El dolor neuropático punzante fue el más frecuente: en la cabeza, se presentó en el 77,6 % de los casos y, en las plantas de los pies, en el 63,2% de las personas. El dolor neuropático quemante fue el segundo más frecuente en todos los sitios, siendo el muscular en otro sitio el más percibido (47,8 % de los participantes). Por último, el 67,2 % de los participantes puntuó entre 5 y 8 la intensidad del dolor en todos los sitios anatómicos. El porcentaje más alto se presentó para el dolor en los músculos de las piernas, en el 41,5 % de los casos, con una puntuación de 5 a 6 en la mayoría de ellos, seguido por el de la planta de los pies, en un 38 % de las personas, con una puntuación de 7 a 8 (cuadro 2).

El 58,7 % de las personas manifestó experimentar cansancio generalizado por más de 12 días, sobre todo durante la fase subaguda, con un 35,9 %. El dolor articular en manos (p: 0,017), tobillos (p: 0,001) y planta de los pies (p: 0,002), fue significativamente mayor en mujeres que en hombres, pero no hubo diferencias estadísticamente significativas en el dolor muscular según el sexo (cuadro 3).

**Cuadro 3:** Prevalencia del dolor según tipo, sitio anatómico y sexo de los pacientes

Sitio anatómico	Sexo				Total (n=368)		χ**2**
	Femenino (n=258)		Masculino (n=110)				
	n	Prevalencia	n	%	n	%	p
Cabeza	50	19,4	17	15,5	67	18,2	0,372
Hombro	78	30,2	36	32,7	114	31	0,636
Codo	78	30,2	34	30,9	112	30,4	0,897
Mano	225	87,2	85	77,3	310	84,2	0,017
Cadera	68	26,4	26	23,6	94	25,5	0,584
Rodilla	191	74	77	70	268	72,8	0,426
Tobillo	192	74,4	63	57,3	255	69,3	0,001
Otras articulaciones	26	10,1	7	6,4	33	9	0,254
Espalda	29	11,2	9	8,2	38	10,3	0,377
Músculos del brazo	47	18,2	20	18,2	67	18,2	0,994
Músculos de las piernas	48	18,6	15	13,6	63	17,1	0,247
Muslos	42	16,3	19	17,3	61	16,6	0,814
Otros músculos	16	6,2	7	6,4	23	6,3	0,953
Planta de los pies	127	49,2	35	31,8	162	44	0,002

## Discusión

Los principales hallazgos del presente análisis coinciden con los de diversos estudios ^14^^-^^19^: en la muestra hubo un mayor porcentaje de mujeres, aunque en la metodología empleada en este se utilizó un muestreo no probabilístico en bola de nieve; asimismo, los pacientes manifestaron haber experimentado los tres síntomas principales descritos para la fiebre de chikungunya (artralgia, mialgia y astenia) ^20^.

Por otra parte, las articulaciones más frecuentemente afectadas por el dolor fueron las de las manos (84,2 %), las rodillas (72,8 %) y los tobillos (69,3 %), lo que coincide con lo reportado en un estudio en el Caribe colombiano ^18^; después de los sitios anatómicos ya mencionados, la planta de los pies fue el sitio más afectado por el dolor (44 %).

La cronicidad de los síntomas de la fiebre de chikungunya se debe a la conjunción de la reacción autoinmunitaria frente a antígenos virales persistentes y a la presencia crónica del virus (o de sus productos) en las células diana, con la consecuente acumulación local de mediadores inflamatorios; por ejemplo, en macacos experimentalmente infectados por el virus del chikungunya se ha observado la presencia de antígenos virales y de ARN viral en órganos linfoides y tejido sinovial meses después de la fase aguda de la infección ^21^; pero, aunque se pueden presentar síntomas atribuibles a la fiebre de chikungunya por más de tres meses, la proporción de personas afectadas disminuye con el tiempo ^16^^,^^18^^-^^20^, lo que corrobora los resultados del presente estudio, en el cual se pudo constatar que el número de personas con dolor en todos los sitios anatómicos bajo estudio disminuyó con cada trimestre evaluado.

El porcentaje de las personas con dolor discontinuo fue similar al de aquellas con dolor continuo, lo que concuerda con los hallazgos de un estudio en Risaralda ^16^, en el cual se reportaron síntomas de rigidez matutina en el 60,5 % de los pacientes mayores de 40 años y en el 45,4 % de los menores de 40 años.

Si bien la intensidad del dolor es un aspecto que no depende solamente de la nocicepción, sino también de factores psicológicos y culturales ^22^, la descrita por los pacientes en este estudio y en el ya mencionado fue similar ^16^, con una mediana de 6 en la misma escala.

Se encontró que el dolor en manos, tobillos y plantas de los pies, significativamente mayor en las mujeres, se asoció con la frecuencia de poliartralgia crónica después de la fiebre de chikungunya y la presencia de rigidez matinal, también significativamente mayor en mujeres según el estudio en Risaralda ^16^.

La principal limitación metodológica de este estudio fue no haber determinado como criterio de exclusión los antecedentes de dolor articular o de otro tipo de dolor en los pacientes antes de presentar la fiebre de chikungunya.
